# Enhanced production of l-sorbose by systematic engineering of dehydrogenases in *Gluconobacter oxydans*

**DOI:** 10.1016/j.synbio.2022.02.008

**Published:** 2022-03-16

**Authors:** Li Liu, Yue Chen, Shiqin Yu, Jian Chen, Jingwen Zhou

**Affiliations:** a National Engineering Laboratory for Cereal Fermentation Technology, Jiangnan University, 1800 Lihu Road, Wuxi, Jiangsu, 214122, China; b School of Biotechnology and Key Laboratory of Industrial Biotechnology, Ministry of Education, Jiangnan University, 1800 Lihu Road, Wuxi, Jiangsu, 214122, China; c Science Center for Future Foods, Jiangnan University, 1800 Lihu Road, Wuxi, Jiangsu, 214122, China; d Jiangsu Provisional Research Center for Bioactive Product Processing Technology, Jiangnan University, 1800 Lihu Road, Wuxi, Jiangsu, 214122, China

**Keywords:** D-sorbitol dehydrogenase, *Gluconobacter oxydans*, l-ascorbic acid, Metabolic engineering

## Abstract

l-Sorbose is an essential intermediate for the industrial production of vitamin C (l-ascorbic acid)*.* However, the formation of fructose and some unknown by-products significantly reduces the conversion ratio of D-sorbitol to l-sorbose. This study aimed to identify the key D-sorbitol dehydrogenases in *Gluconobacter oxydans* WSH-003 by gene knockout. Then, a total of 38 dehydrogenases were knocked out in *G. oxydans* WSH-003, and 23 dehydrogenase-deficient strains could increase l-sorbose production. *G. oxydans*-30, wherein a pyrroloquinoline quinone-dependent glucose dehydrogenase was deleted, showed a significant reduction of a by-product with the extension of fermentation time. In addition, the highest conversion ratio of 99.60% was achieved in *G. oxydans* MD-16, in which 16 different types of dehydrogenases were inactivated consecutively. Finally, the gene *vhb* encoding hemoglobin was introduced into the strain. The titer of l-sorbose was 298.61 g/L in a 5-L bioreactor. The results showed that the systematic engineering of dehydrogenase could significantly enhance the production of l-sorbose.

## 1 Introduction

l-sorbose is an essential precursor for the industrial fermentation production of 2-keto-l-gulonic acid (2-KLG), which is the direct precursor of l-ascorbic acid (vitamin C) [1,2]. The most used industrial method for producing l-sorbose is the conversion of D-sorbitol by *Gluconobacter* or *Acetobacter* species [3,4] in which membrane-bound D-sorbitol dehydrogenases are responsible for this enzymatic conversion. The catalytic activity center of membrane-bound D-sorbitol dehydrogenases is commonly exposed to the periplasmic space; therefore, the l-sorbose is generally produced in the periplasm and subsequently secreted into the culture broth [5,6]. In the classical vitamin C production process, the l-sorbose produced by *Gluconobacter* spp. was further converted into 2-KLG via a mixed fermentation of *Ketogulonicigenium vulgare* and *Bacillus megaterium* [7]*,* and the resulting product 2-KLG was chemically esterified for vitamin C production [1,8].

*Gluconobacter* belongs to the family of *Acetobacteraceae* and is unsurpassed by other organisms in the capacity of incompletely oxidizing a great variety of carbohydrates, alcohols, and related compounds [4,9]. It can oxidize many substrates regio-selectively, hence is widely used for the industrial production of l-sorbose, 6-amino-l-sorbose, d-gluconic acid, and keto-gluconic acids [4,9]. l-sorbose is an essential substrate in vitamin C production, and mainly two types of membrane-bound D-sorbitol dehydrogenase are involved in the oxidation of D-sorbitol. One is pyrroloquinoline quinone (PQQ) dependent D-sorbitol dehydrogenase with two subunits (sldB and sldA), and the other one is FAD dependent D-sorbitol dehydrogenase with three subunits (sldS, sldL, and sldC) [10,11]. In addition, some cytoplasmic D-sorbitol dehydrogenases or xylitol dehydrogenases in *G. oxydans* mainly catalyze D-sorbitol to form d-fructose for cell growth [12,13]. Some special NADP^+^/NAD^+^-dependent D-sorbitol dehydrogenases from *G. oxydans* G624 could also catalyze D-sorbitol to form l-sorbose efficiently [14]. The membrane-bound D-sorbitol dehydrogenases are usually considered as the main enzymes responsible for catalyzing D-sorbitol into l-sorbose.

Genome deletion is expected to obtain genome-simplified chassis cells with rich application potential. Some studies on the simplification of the genomes of model microorganisms have been reported, including *B. subtilis*, *Escherichia coli* [15], *Corynebacterium glutamicum* [16], and *Streptomyces avermitilis* [17]. For *G. oxydans*, studies were mainly performed to identify the function of some genes by gene knockout, such as the gene cluster for the biosynthesis of PQQ [18] and the characterization of membrane-bound dehydrogenases from *G. oxydans* 621H [19]. Knockout of eight membrane-bound dehydrogenases in *G. oxydans* 621H could improve the titer and conversion rate of l-erythrulose to 242 g/L and 99%, respectively, in a dissolved oxygen (DO)-controlled stirred-tank bioreactor [20]. Thus, deleting enzymes unrelated to the l-sorbose formation may enhance the l-sorbose production by avoiding unnecessary energy consumption and providing limited membrane space to the most needed membrane-bound D-sorbitol dehydrogenase [21].

In this study, the critical membrane-bound D-sorbitol dehydrogenases in *G. oxydans* WSH-003 were identified by gene knockout. The genes of *sldBA1* and *sldSLC* encode major membrane-bound D-sorbitol dehydrogenases that catalyze the conversion of D-sorbitol into l-sorbose. On the contrary, the gene of *sldBA2* showed no catalytic activity for D-sorbitol, although it had high homology with *sldBA1*. After identifying the key D-sorbitol dehydrogenases of *G. oxydans* WSH-003, 38 predicted dehydrogenases unrelated to the synthesis of l-sorbose were knocked out. The results showed that 23 dehydrogenase-deficient strains could increase l-sorbose production and *G. oxydans*-30, a membrane-bound glucose dehydrogenase (mGDH)-deficient strain of them could significantly decrease a by-product in the fermentation broth with the extension of fermentation time. At the same time, *G. oxydans* MD-16, in which 16 kinds of dehydrogenases were knocked out consecutively, had the highest titer of l-sorbose (149.46 g/L) with a conversion rate of 99.60% in shake flasks. Besides, the overexpression of the gene *vhb* encoding *Vitreoscilla* hemoglobin (VHb) in *G. oxydans* MD-16, scaling up in a 5-L fermenter, the titer of l-sorbose reached 298.61 g/L in 300 g/L D-sorbitol medium.

## 2 Materials and methods

### 2.1 Strains, plasmids, and culture conditions

*G. oxydans* WSH-003 was obtained from Jiangsu Jiangshan Pharmaceutical Co., Ltd., and was sequenced in a previous study (GenBank Accession No. AHKI00000000.1) [22]. *E. coli* JM109 (*E. coli* JM109) was purchased from Novagen (Darmstadt, Germany) and used as a host for plasmid construction and amplification. The pMD19-T simple vector was purchased from TaKaRa (Dalian, China). The *E. coli* JM109 strains were cultivated in Luria-Bertani (LB) medium at 37 °C and 220 rpm. When required, tetracycline was added to a final concentration of 20 μg/mL. The *G. oxydans* strains were cultivated in D-sorbitol medium (80 g/L D-sorbitol and 10 g/L yeast extract) at 30 °C and 220 rpm. When required, fluorouracil (FU), kanamycin, cefoxitin, and tetracycline were added to a final concentration of 300 μg/mL, 50 μg/mL, 50 μg/mL, and 20 μg/mL, respectively.

### 2.2 Gene deletions and construction of recombinant strains

For knocking out dehydrogenases, the upstream and downstream homologous arms of dehydrogenases were amplified by PCR from the *G. oxydans* WSH-003 genome. The kanamycin resistance gene (kana) or kana-upp fusion segments was obtained by PCR amplification, and it was fused with the upstream and downstream homologous arms of dehydrogenases with resistance gene. Fragments (DHS-kana-DHX and DHS-kana-upp-DHX) corresponding to related dehydrogenases was obtained. Strains in which dehydrogenases was knocked out were generated by integrating the obtained homologous recombinant fragments into the *G. oxydans* WSH-003 parental strain. Membrane-bound D-sorbitol dehydrogenase genes of *G. oxydans* WSH-003 were deleted consecutively using the counter-selection marker of *upp* gene. D-sorbitol dehydrogenase knockout strains are listed in Table 1. The primers used to construct the D-sorbitol dehydrogenase knockout strains are given in Supplementary Table (Table S1). Dehydrogenases of unknown function of *G. oxydans* WSH-003 were deleted using the *kana* gene. Dehydrogenase of unknown function knockout strains are listed in Table 1. The primers used to construct the dehydrogenase knockout strains are given in Supplementary Table (Table S1). Dehydrogenase genes of *G. oxydans* WSH-003 were deleted consecutively using the counter-selection marker of the *upp* gene. Dehydrogenase combination knockout strains are listed in Table 1. The primers used to construct the combination knockout strain vectors are given in Supplementary Table (Table S1). All mutants were verified by polymerase chain reaction and sequencing.

**Table 1:** Strains and plasmids.

Strains or plasmids	Characteristics	Sources
*G. oxydans* Δ*upp*	*upp*	This study
*G. oxydans*-A	*upp* and *sldBA1*	This study
*G. oxydans*-B	*upp* and *sldBA2*	This study
*G. oxydans*-C	*upp* and *sldSLC*	This study
*G. oxydans*-D	*upp*, *sldBA1* and *sldBA2*	This study
*G. oxydans*-E	*upp*, *sldBA1* and *sldSLC*	This study
*G. oxydans-*F	*upp*, *sldBA2* and *sldSLC*	This study
*G. oxydans*-G	*upp*, *sldBA1*, *sldBA2* and *sldSLC*	This study
*G. oxydans*-E	*upp*, *sldBA1* and *sldSLC*	This study
*G. oxydans*-1	alcohol DH 2	This study
*G. oxydans*-2	D-arabitol DH	This study
*G. oxydans*-3	gluconate 5-DH	This study
*G. oxydan* −4	lactate DH	This study
*G. oxydans*-5	l-idonate 5-DH	This study
*G. oxydans*-6	NAD(P)H DH (quinone)	This study
*G. oxydans*-7	NAD-dependent xylitol DH 2	This study
*G. oxydans*-8	2-hydroxyacid DH	This study
*G. oxydans*-9	alcohol DH 3	This study
*G. oxydans*-10	alcohol DH 4	This study
*G. oxydans*-11	aldehyde DH	This study
*G. oxydans*-12	sorbosone DH	This study
*G. oxydans*-13	d-lactate DH	This study
*G. oxydans*-14	glucose-6-phosphate DH	This study
*G. oxydans*-15	isocitrate DH	This study
*G. oxydans*-16	l-sorbose 1-DH	This study
*G. oxydans*-17	NAD(P)H DH (quinone) 2	This study
*G. oxydans*-18	NAD-dependent xylitol DH	This study
*G. oxydans*-19	NADH DH (ubiquinone)	This study
*G. oxydans*-20	PQQ-containing DH 2	Failed
*G. oxydans*-21	short chain DH	This study
*G. oxydans*-22	short-chain DH 3	This study
*G. oxydans*-23	zinc-dependent alcohol DH	This study
*G. oxydans*-24	zinc-type alcohol DH	This study
*G. oxydans*-25	gluconate2-DH	This study
*G. oxydans*-26	glucose-DH	Failed
*G. oxydans*-27	NADH DHtypeII2	Failed
*G. oxydans*-28	NADH DH type II	Failed
*G. oxydans*-29	Aldehyde DH-like protein	This study
*G. oxydans*-30	Glucose DH2	This study
*G. oxydans*-31	Mannitol DH	This study
*G. oxydans*-32	glucose DH(PQQ)	This study
*G. oxydans*-33	Hydroxyacid DH	This study
*G. oxydans*-34	short-chain DH reductase 2	This study
*G. oxydans*-35	short-chain DH reductase	This study
*G. oxydans*-36	aldehyde DH 2	Failed
*G. oxydans*-37	alcohol DH	This study
*G. oxydans*-38	aldehyde DH (NAD(+))	This study
*G. oxydans*-39	aldehyde DH 3	This study
*G. oxydans*-40	alpha-hydroxy-acid oxidizing enzyme	Failed
*G. oxydans*-41	glucose DH	This study
*G. oxydans*-42	NADH-dependent alcohol DH	This study
*G. oxydans*-43	NADH DH (quinone)	This study
*G. oxydans*-44	PQQ-dependent DH 3	This study
*G. oxydans* MD-1	*G. oxydans* Δ *upp* and l-sorbosone DH	This study
*G. oxydans* MD-2	MD-1ΔGlucose DH2	This study
*G. oxydans* MD-3	MD-2ΔNAD-dependent xylitol DH	This study
*G. oxydans* MD-4	MD-3ΔNAD-dependent xylitol DH 2	This study
*G. oxydans* MD-5	MD-4Δ gluconate 5-DH	This study
*G. oxydans* MD-6	MD-5 Δ sldSLC	This study
*G. oxydans* MD-7	MD-6 ΔPTS	This study
*G. oxydans* MD-8	MD-7Δglucose DH	This study
*G. oxydans* MD-9	MD-8 ΔMannitol DH	This study
*G. oxydans* MD-10	MD-9 ΔPQQ-dependent DH 3	This study
*G. oxydans* MD-11	MD-10 Δalcohol DH	This study
*G. oxydans* MD-12	MD-11Δaldehyde DH 3	This study
*G. oxydans* MD-13	MD-12Δaldehyde DH (NAD(+))	This study
*G. oxydans* MD-14	MD-13Δgluconate2-DH	This study
*G. oxydans* MD-15	MD-14Δaldehyde DH	This study
*G. oxydans* MD-16	MD-15Δaldehyde DH	This study
*G. oxydans* MD-17	MD-16ΔPQQ-dependent alcohol DH	This study
*G. oxydans* MD-18	MD-17Δcarboxylate DH	This study
*G. oxydans* MD-19	MD-18Δmembrane-bound glucose DH	This study
*G. oxydans* MD-20	MD-19Δaltronate DH	This study
*G. oxydans* MD-21	MD-20Δaldehyde dehydrogenas2	This study
*G. oxydans* MD-22	MD-21Δalcohol DH	This study
*G. oxydans* MD-23	MD-22Δmolybdopterin DH	This study
*G. oxydans* MD-24	MD-23ΔL-Sorbose Reductase	This study
*G. oxydans* MD-25	MD-24ΔD-arabitol DH	This study
*G. oxydans* MD-26	MD-25Δalcohol DH	This study
*G. oxydans* MD-27	MD-26Δalcohol DH	This study
**Plasmids**		
p13-tet	shuttle vector, Tet^R^	[23]
p13-tet-P_2703_-*vhb*	P_2703_ promoter with *vhb*, Tet^R^	This study
p13-tet-P_vhb_-*vhb*	P_vhb_ promoter with *vhb*, Tet^R^	This study
p13-tet-P_2703_-Tat-*vhb*	P_2703_ promoter with tat-*vhb*, Tet^R^	This study
p13-tet-P_vhb_- Tat-*vhb*	P_vhb_ promoter with tat-*vhb*, Tet^R^	This study

### 2.3 Overexpression of *vhb* in *G. oxydans*

A plasmid containing the *vhb* gene driven by the P_2703_ promoter or P_vhb_ promoter was constructed to express *vhb* in *G. oxydans* [24]. The *vhb* gene with the promoter was synthesized by Sangon (Shanghai, China). The VHb was fused to the twin-arginine signal peptide to export it into the periplasm [25]. The plasmids and primers are listed in Table 1 and Supplementary Table (Table S1), respectively. All plasmids constructed were transformed into competent *E. coli* JM109, and transformants were selected on LB medium plates containing 20 μg/mL tetracycline. Transformation of *G. oxydans* was carried out using electroporation by the methods described previously [26].

### 2.4 Production of l-sorbose by *G. oxydans* in flasks

*G. oxydans* WSH-003 and dehydrogenase knockout strains preserved in −80 °C ultra-low temperature refrigerator were streaked on D-sorbitol medium plates and cultured for 48 h at 30 °C. One single colony was picked up and inoculated into 250-mL flasks with 25 mL of D-sorbitol medium at 30 °C for 48 h, and then transferred to 25 mL of fermentation medium (D-sorbitol, 150 g/L; yeast extract, 10 g/L) in a 250-mL flask. The inoculation volume was 10% (v/v) and cultivations were performed at 30 °C. Tetracycline (20 mg/L), kanamycin (50 mg/L), or/and cephalosporins (50 mg/L) were added when necessary.

### 2.5 Production of l-sorbose by *G. oxydans* in a 5-L bioreactor

The batch fermentation process was performed to optimize the production of l-sorbose in a 5-L bioreactor system (T&J Bioengineering, Shanghai, China) filled with 3 L of fermentation medium (D-sorbitol, 300 g/L; yeast extract, 10 g/L). A 10% (*v*/*v*) inoculum of *G. oxydans* was added to the bioreactor and incubated at 30 °C at 400 rpm. The aeration rate was controlled at 1 vvm.

### 2.6 Analysis procedures

D-sorbitol and l-sorbose levels in the fermentation broth were determined by high-performance liquid chromatography (HPLC) (Shimadzu, Kyoto, Japan) using an Aminex HPX-87H column (Bio-Rad, CA, USA) at 35 °C with a flow rate of 0.5 mL/min and 5 mmol/L H_2_SO_4_ as the eluent. The refractive index detector was selected for detection.

## 3 Results and discussion

### 3.1 Identification of the key D-sorbitol dehydrogenases of *G. oxydans* WSH-003

**Fig. 1:**
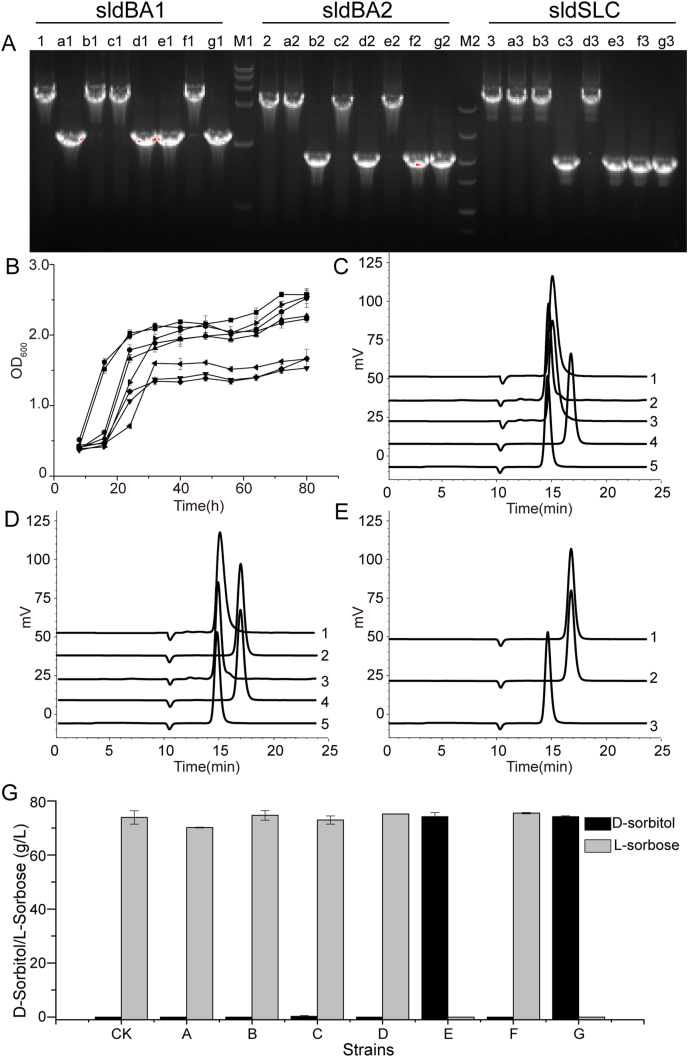
Identification of key D-sorbitol dehydrogenase in *G. oxydans*. (A) Agarose gel electrophoresis validation of *G. oxydans*. WSH-003 D-sorbitol dehydrogenase knockout strains. M1, DL15000 DNA marker; M2, DL5000 DNA marker; 1-g1: Validation of *sldBA1* in *G. oxydans-*Δ*upp*, *G. oxydans-*A, *G. oxydans-*B, *G. oxydans-*C, *G. oxydans-*D, *G. oxydans-*E*, G. oxydans-*F, and *G. oxydans-*G*,* respectively; 2-g2: Validation of *sldBA2* in *G. oxydans-*Δ*upp*, *G. oxydans-*A, *G. oxydans-*B, *G. oxydans-*C, *G. oxydans-*D, *G. oxydans-*E, *G. oxydans-*F, and *G. oxydans-*G*,* respectively; 3-g3: Validation of *sldSLC* in *G. oxydans-*Δ*upp*, *G. oxydans-*A, *G. oxydans-*B, *G. oxydans-*C, *G. oxydans-*D, *G. oxydans-*E, *G. oxydans-*F, and *G. oxydans-*G*,* respectively. (B) Growth curves of different D-sorbitol dehydrogenase-deficient strains. Square, *G. oxydans* Δ*upp*; circle, *G. oxydans*-A; up-triangle, *G. oxydans*-B; down-triangle, *G. oxydans*-C; rhombus, *G. oxydans*-D; left triangle, *G. oxydans*-E; right triangle, *G. oxydans*-F; pentagon, *G. oxydans*-G. (C) HPLC results of one sorbitol dehydrogenase–deficient strains. 1, *G. oxydans*-A; 2, *G. oxydans*-B; 3, *G. oxydans*-C; 4, D-sorbitol; 5, l-sorbose. (D) HPLC results of two sorbitol dehydrogenase–deficient strains. 1, *G. oxydans*-D; 2, *G. oxydans*-E; 3, *G. oxydans*-F; 4, D-sorbitol; 5, l-sorbose. (E) HPLC results of three sorbitol dehydrogenase−deficient strains. 1, *G. oxydans*-G; 2, D-sorbitol; 3, l-sorbose. (G) Comparison of l-sorbose titer of different D-sorbitol dehydrogenase-deficient strains. CK: *G. oxydans-*Δ*upp*, A: *G. oxydans-*A, B: *G. oxydans-*B, C: *G. oxydans-*C, D: *G. oxydans-*D, E: *G. oxydans-*E, F: *G. oxydans-*F, G: *G. oxydans-*G.

The membrane-bound D-sorbitol dehydrogenase clusters are sldBA1, sldBA2, and sldSLC from *G*, *oxydans* WSH-003. However, the genes with the function of conversion of D-sorbitol into l-sorbose have not been studied. The knockout strains of different D-sorbitol dehydrogenases genes (*sldBA1*, *sldBA2*, and *sldSLC*) from *G. oxydans* WSH-003 are shown in Table 1. Verification of these strains with D-sorbitol dehydrogenase deficiency is shown in Fig. 1A. After cultivating these strains in D-sorbitol medium, the result showed no significant change in their growth compared with the wild-type strain (Fig. 1B). Among these, the growth of *G. oxydans*-C, *G. oxydans*-D, and *G. oxydans*-E decreased slightly; *G. oxydans*-G, which was deficient in all three D-sorbitol dehydrogenases, could also grow well in the D-sorbitol medium. *G. oxydans*-A, *G. oxydans*-B, and *G. oxydans*-C had no significant effects on the conversion of D-sorbitol into l-sorbose (Fig. 1C), and the l-sorbose titer were 70.17 g/L, 74.69 g/L and 72.94 g/L (Fig. 1G). However, *G. oxydans*-E that knocked out the genes of *sldBA1* and *sldSLC* could not form l-sorbose from D-sorbitol (Fig. 1D). There was not l-sorbose produced in the fermentation broth, and still 74.16 g/L D-sorbitol left in the fermentation broth (Fig. 1G). The fermentation results of *G. oxydans*-G were the same as that of *G. oxydans*-E (Fig. 1E), and there was still 74.19 g/L D-sorbitol left in the fermentation broth (Fig. 1G).

l-sorbose is an essential intermediate for manufacturing high value-added products, such as vitamin C [2] and l-tagatose [27]. In the fermentation of l-sorbose, the highly active D-sorbitol dehydrogenase is essential. Although *in vitro* characterization of D-sorbitol dehydrogenase was conducted by exogenous expression of D-sorbitol dehydrogenase in *E. coli* [14] or direct isolation and identification of D-sorbitol dehydrogenase from *G. oxydans* [11], these *in vitro* results could not directly represent their *in vivo* production capacity in *G. oxydans* because membrane-bound dehydrogenases require the channeling of electrons into the respiratory chain [28]. Thus, in the present study, the critical D-sorbitol dehydrogenase of *G. oxydans* WSH-003 was verified by gene knockout, demonstrating that D-sorbitol dehydrogenases *sldBA1* and *sldSLC* were key enzymes for l-sorbose formation, and one of them was sufficient to catalyze D-sorbitol to l-sorbose (Fig. 1G). Additionally, the resulting *G. oxydans*-G and *G. oxydans*-E, which lost the capacity lost the capacity to convert D-sorbitol to l-sorbose, may be used as a platform strain applied in the screening of high-activity D-sorbitol dehydrogenase in future studies.

### 3.2 Effects of dehydrogenase gene knockout on l-sorbose production

**Fig. 2:**
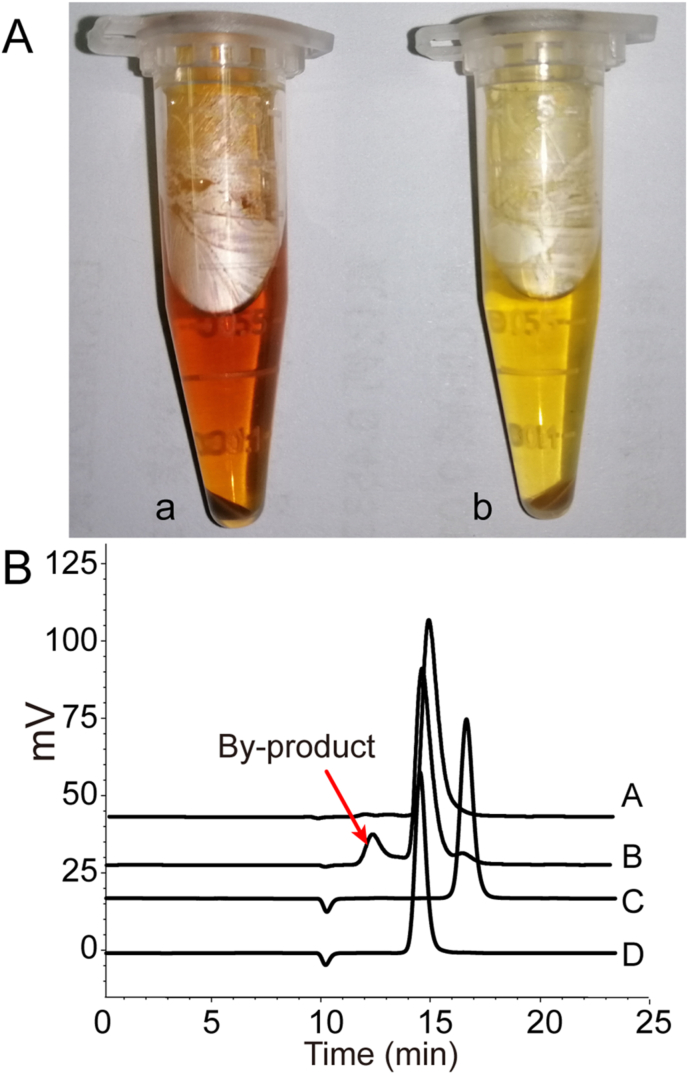
**Fermentation broth of different *G. oxydans* and their HPLC results.** (A) Color of *G. oxydans* fermentation broth. a, *G. oxydans* WSH-003; b, *G. oxydans*-30. (B) HPLC results of *G. oxydans* WSH-003 and *G. oxydans*-30. A, *G. oxydans*-30; B, *G. oxydans* WSH-003; C, D-sorbitol; D, l-sorbose. (For interpretation of the references to color in this figure legend, the reader is referred to the Web version of this article.)

A total of 44 dehydrogenases of *G. oxydans* WSH-003 were selected to be knocked out, and dehydrogenase single knockout strain was used to verify the effect of dehydrogenase knockout on the production of l-sorbose. Among the 44 dehydrogenases, 6 dehydrogenases (PQQ-containing dehydrogenase 2, glucose dehydrogenase, NADH dehydrogenase type II2, NADH dehydrogenase type II, glucose dehydrogenase, and aldehyde dehydrogenase 2) could not be successfully knocked out in *G. oxydans* WSH-003, indicating that they might be essential genes. Finally, 38 dehydrogenase genes were knocked out successfully. Among the 38 dehydrogenase-deficient strains, 23 dehydrogenase-deficient strains could increase l-sorbose production. Compared with *G. oxydans* WSH-003 (136.95 g/L), the l-sorbose production with *G. oxydans*-12, *G. oxydans*-18, and *G. oxydans*-31 increased obviously, reaching 144.89 g/L, 144.14 g/L, and 144.77 g/L, respectively (Table 2). Noticeably, the fermentation broth color of *G. oxydans*-30 was significantly different from that of *G. oxydans* WSH-003 with the extension of fermentation time (Fig. 2A). At the same time, an unknown by-product was obviously decreased in the culture broth of *G. oxydans*-30 using HPLC analysis (Fig. 2B). This by-product might be a reason why the strain could not reached a higher conversion rate.

**Table 2:** Production of l-sorbose by various dehydrogenase knock-out strains.

Strains	l-Sorbose (g/L)	OD_600_	l-Sorbose titer per OD_600_
WSH-003	136.95 ± 3.04	2.96 ± 0.12	46.23
*G. oxydans*-1	137.43 ± 3.50	2.95 ± 0.01	46.61
*G. oxydans*-2	134.53 ± 5.27	2.94 ± 0.09	45.70
*G. oxydans*-3	136.85 ± 4.74	2.99 ± 0.04	45.76
*G. oxydan*-4	136.66 ± 4.11	3.10 ± 0.02	44.04
*G. oxydans*-5	141.74 ± 4.03	2.75 ± 0.06	51.59
*G. oxydans*-6	136.27 ± 4.83	3.51 ± 0.05	38.82
*G. oxydans*-7	139.13 ± 0.62	2.88 ± 0.03	48.30
*G. oxydans*-8	140.04 ± 3.50	2.71 ± 0.04	51.69
*G. oxydans*-9	136.79 ± 4.82	3.27 ± 0.09	41.82
*G. oxydans*-10	135.09 ± 3.81	2.69 ± 0.07	60.31
*G. oxydans*-11	135.93 ± 4.69	2.90 ± 0.02	46.82
*G. oxydans*-12	144.89 ± 1.89	2.73 ± 0.10	63.75
*G. oxydans*-13	139.69 ± 5.07	3.29 ± 0.02	42.50
*G. oxydans*-14	139.74 ± 4.66	2.92 ± 0.03	47.87
*G. oxydans*-15	135.90 ± 4.08	2.20 ± 0.04	61.73
*G. oxydans*-16	138.91 ± 4.10	3.56 ± 0.04	38.99
*G. oxydans*-17	140.76 ± 2.21	2.83 ± 0.03	49.74
*G. oxydans*-18	144.14 ± 1.37	2.26 ± 0.07	65.52
*G. oxydans*-19	141.86 ± 4.55	3.34 ± 0.03	43.23
*G. oxydans*-21	139.97 ± 0.60	2.76 ± 0.05	52.92
*G. oxydans*-22	143.89 ± 5.34	2.71 ± 0.01	53.08
*G. oxydans*-23	139.55 ± 0.77	2.19 ± 0.03	65.24
*G. oxydans*-24	140.85 ± 0.80	2.25 ± 0.02	62.73
*G. oxydans*-25	139.72 ± 1.20	2.93 ± 0.01	47.69
*G. oxydans*-29	141.94 ± 1.86	3.27 ± 0.06	43.47
*G. oxydans*-30	138.67 ± 3.15	3.29 ± 0.16	42.17
*G. oxydans*-31	144.77 ± 2.60	2.81 ± 0.08	51.54
*G. oxydans*-32	138.75 ± 3.38	3.30 ± 0.02	42.06
*G. oxydans*-33	137.03 ± 0.52	3.27 ± 0.02	41.90
*G. oxydans*-34	136.44 ± 5.48	2.70 ± 0.14	51.67
*G. oxydans*-35	139.29 ± 0.53	3.21 ± 0.04	43.35
*G. oxydans*-37	143.15 ± 0.49	3.30 ± 0.09	42.12
*G. oxydans*-38	142.20 ± 1.80	3.08 ± 0.10	44.77
*G. oxydans*-39	136.53 ± 0.23	2.73 ± 0.06	50.01
*G. oxydans*-41	140.47 ± 0.75	3.00 ± 0.01	46.86
*G. oxydans*-42	136.21 ± 0.31	3.10 ± 0.03	43.95
*G. oxydans*-43	134.85 ± 2.59	2.82 ± 0.02	47.77
*G. oxydans*-44	139.89 ± 0.31	2.64 ± 0.01	53.04

The dehydrogenase promiscuity may allow for the use of cost-effective feedstock as substrates; however, these dehydrogenases may increase the formation of by-products and influence the yield and the downstream purification process. Therefore, reducing by-product formation through gene knockout is a valuable tool to demonstrate the function of a specific gene [[29], [30], [31]]. This study found that the number of by-products in the fermentation broth significantly reduced after membrane-bound glucose dehydrogenase was knocked out (Fig. 2). According to their protein sequences, these enzymes belonged to the quinoprotein GDH (EC 1.1.5.2) with PQQ as a cofactor in gram-negative bacteria [[32], [33], [34]]. The substrate spectra of GDH were broad, including d-galactose, d-mannose, d-xylose, l-arabinose, maltose, and d-allose [28]. The newly discovered membrane-bound glucose dehydrogenase with catalytic activity in l-sorbose conversion has expanded the substrate spectrum of the glucose dehydrogenase family. It can be used to produce other valuable products.

### 3.3 Effects of combinatorial knockout of dehydrogenase genes on l-sorbose production

The aforementioned results showed that some *G. oxydans* WSH-003 dehydrogenase−deficient strains increased the production of l-sorbose and reduced the formation of by-products. Therefore, a total of 27 genes of *G. oxydans* WSH-003 were knocked out by consecutively deleting one gene after the other using the *upp* gene as counter-selection markers [35]. Among the 27 *G. oxydans* WSH-003 dehydrogenase−deficient strains, the highest titer and yield of the strain *G. oxydans* MD-16 was 149.46 g/L and 99.60%, respectively, which increased by 6.54% compared with that of *G. oxydans* WSH-003 (93.06%) (Table 3). Regarding the growth of *G. oxydans* WSH-003 dehydrogenase–deficient strains, *G. oxydans* MD-1, *G. oxydans* MD-2, *G. oxydans* MD-3, *G. oxydans* MD-4, *G. oxydans* MD-5, and *G. oxydans* MD-15 grew better than *G. oxydans* Δ*upp*. However, the final OD_600_ of *G. oxydans* MD-26 and *G. oxydans* MD-27 were just 1.14 and 1.10, respectively, which was reduced by more than 50% and could not be further engineered for gene knockout (Table 3).

**Table 3:** Comparison of multi-deletion strains for l-sorbose production.

Strains	l-Sorbose (g/L)	OD_600_	l-Sorbose titer per OD_600_
*G. oxydans*-Δ*upp*	136.9 ± 0.38	2.84 ± 0.03	48.20
*G. oxydans* MD-1	144.43 ± 2.09	3.23 ± 0.01	44.72
*G. oxydans* MD-2	147.65 ± 4.14	3.29 ± 0.25	44.88
*G. oxydans* MD-3	144.04 ± 0.11	3.18 ± 0.06	45.30
*G. oxydans* MD-4	148.66 ± 0.76	3.29 ± 0.13	45.19
*G. oxydans* MD-5	144.47 ± 1.42	3.36 ± 0.49	43.00
*G. oxydans* MD-6	144.43 ± 0.79	2.87 ± 0.08	50.32
*G. oxydans* MD-7	145.89 ± 8.66	2.98 ± 0.07	48.96
*G. oxydans* MD-8	140.42 ± 0.87	2.94 ± 0.02	47.76
*G. oxydans* MD-9	148.55 ± 3.98	2.88 ± 0.19	51.58
*G. oxydans* MD-10	144.83 ± 0.74	2.85 ± 0.02	50.82
*G. oxydans* MD-11	147.61 ± 3.84	2.94 ± 0.01	50.21
*G. oxydans* MD-12	144.52 ± 0.07	2.96 ± 0.01	48.82
*G. oxydans* MD-13	144.25 ± 0.27	2.83 ± 0.11	50.97
*G. oxydans* MD-14	145.53 ± 2.53	2.46 ± 0.09	59.16
*G. oxydans* MD-15	141.11 ± 5.61	3.40 ± 0.11	41.50
*G. oxydans* MD-16	149.46 ± 1.53	2.80 ± 0.09	53.38
*G. oxydans* MD-17	144.22 ± 7.44	2.62 ± 0.03	55.05
*G. oxydans* MD-18	144.43 ± 0.60	2.66 ± 0.18	54.30
*G. oxydans* MD-19	133.12 ± 1.45	2.24 ± 0.10	59.43
*G. oxydans* MD-20	134.01 ± 0.27	2.10 ± 0.03	63.81
*G. oxydans* MD-21	137.33 ± 1.28	2.17 ± 0.06	63.29
*G. oxydans* MD-22	132.68 ± 0.15	1.67 ± 0.01	79.45
*G. oxydans* MD-23	133.86 ± 1.10	1.49 ± 0.10	89.84
*G. oxydans* MD-24	135.10 ± 0.38	1.12 ± 0.04	120.63
*G. oxydans* MD-25	135.43 ± 0.09	1.83 ± 0.16	74.01
*G. oxydans* MD-26	138.65 ± 4.14	1.14 ± 0.04	121.62
*G. oxydans* MD-27	135.04 ± 0.11	1.10 ± 0.05	122.76

In some previous studies, l-sorbose production was enhanced by the overexpression of *sldBA1* with a newly identified strong promoter [3,36]. However, these methods did not increase the conversion rate of D-sorbitol to l-sorbose and reduce by-products in the fermentation process. Thus, gene knockout was necessary to decrease or eliminate the formation of by-products and improve substrate conversion; for example, the cadaverine production increased threefold with the deletion of some genes [37]. The present study showed that deletion of dehydrogenases could indeed improve the conversion rate of D-sorbitol to l-sorbose and reduce the formation of by-products. The conversion rate of the strain *G. oxydans* MD-16 reached up to 99.60%, close to the theoretical conversion rate of D-sorbitol to l-sorbose.

### 3.4 Effects of *vhb* expression on l-sorbose production

**Fig. 3:**
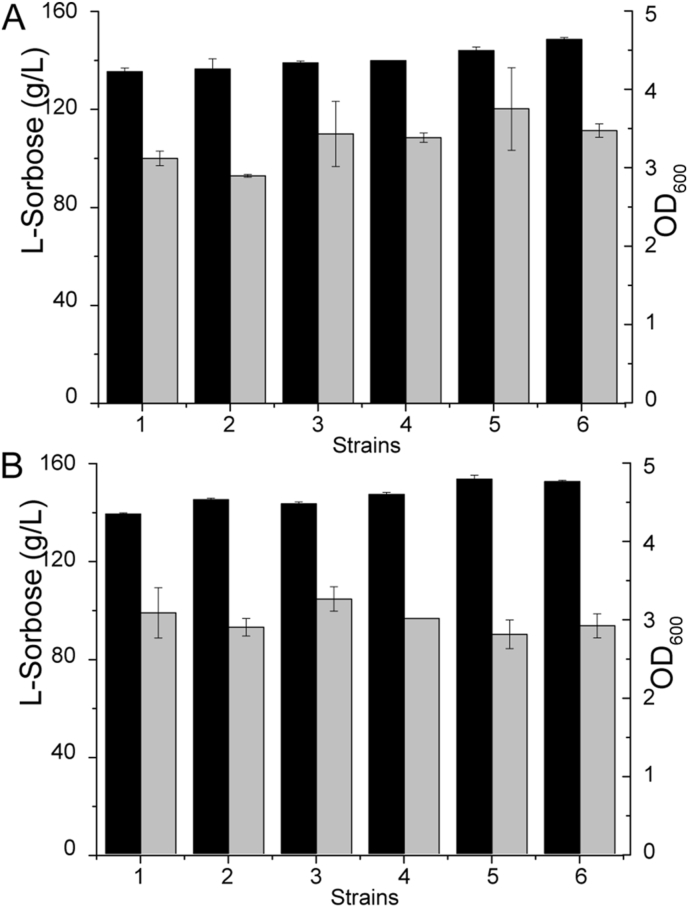
**Effects of VHb expression on****l****-sorbose production in shaking flasks.** (A) Effect of intracellular *vhb* overexpression on cell growth and l-sorbose production. 1, *G. oxydans* WSH-003 with p13-tet; 2, *G. oxydans* WSH-003 with p13-tet-P_2703_-*vhb*, 3, *G. oxydans* WSH-003 with p13-tet-P_vhb_-*vhb*; 4, *G. oxydans* MD-16 with p13-tet; 5, *G. oxydans* MD-16 with p13-tet-P_2703_-*vhb*; 6, *G. oxydans* MD-16 with p13-tet-P_vhb_-*vhb*. (B) Effect of periplasmic *vhb* overexpression on cell growth and l-sorbose production. 1, *G. oxydans* WSH-003 with p13-tet; 2, *G. oxydans* WSH-003 with p13-tet-P_2703_-Tat-*vhb*; 3, *G. oxydans* WSH-003 with p13-tet-P_vhb_-Tat-*vhb*; 4, *G. oxydans* MD-16 with p13-tet; 5, *G. oxydans* MD-16 with p13-tet-P_2703_-Tat-*vhb*; 6, *G. oxydans* MD-16 with p13-tet-P_vhb_-Tat-*vhb*. Gray columns indicate the OD_600_ value, while black columns indicate the production of l-sorbose.

During cell cultivation, the oxygen demand is extremely high because *G. oxydans–*mediated incomplete oxidation of D-sorbitol is closely coupled to the cellular respiration chain [38]. The intracellular and periplasmic *vhb* expression systems were constructed to improve the oxygen utilization efficiency. The results showed that both the final OD_600_ values of *G. oxydans* WSH-003 and *G. oxydans* MD-16 expressing *vhb* were about 3, and the expression of hemoglobin had no obvious effect on cell growth. Besides, the *G. oxydans* MD-16 with p13-tet-P_2703_-Tat-*vhb* had the highest yield of l-sorbose (149.58 g/L), with the conversion ratio of 99.7% (Fig. 3A). The cell membrane imposed limits on the transfer of oxygen to intracellular VHb, and the twin-arginine translocase (Tat) pathway was to export active VHb into the periplasm [25]. The effect of VHb expression in the periplasm was further identified. The results showed that that overexpression of VHb in the periplasm space did not obviously affect the cell growth in shake flask fermentation and could slightly improve the titer of l-sorbose to 149.82 g/L with a conversion ratio of 99.88% (Fig. 3B).

Effective control of DO levels is essential to ensure that microbes maintain high cell densities during the fermentation process, which requires a constant increase in oxygen supply [39]. So far, various solutions have been investigated to increase the DO of the fermentation broth, including increasing agitation speed and oxygen partial pressure, using pure oxygen, or completely rebuilding fermentation vessels [[40], [41], [42]]. However, the aforementioned methods were usually expensive or led to cell damage. Alternatively, the overexpression of *Vitreoscilla* hemoglobin in microorganisms was used to improve cell growth and production [[43], [44], [45]]. In this study, the overexpression of hemoglobin VHb from *Vitreoscilla* increased oxygen availability and improved l-sorbose production without affecting cell growth (Fig. 3).

### 3.5 l-sorbose production in the 5-L bioreactor

**Fig. 4:**
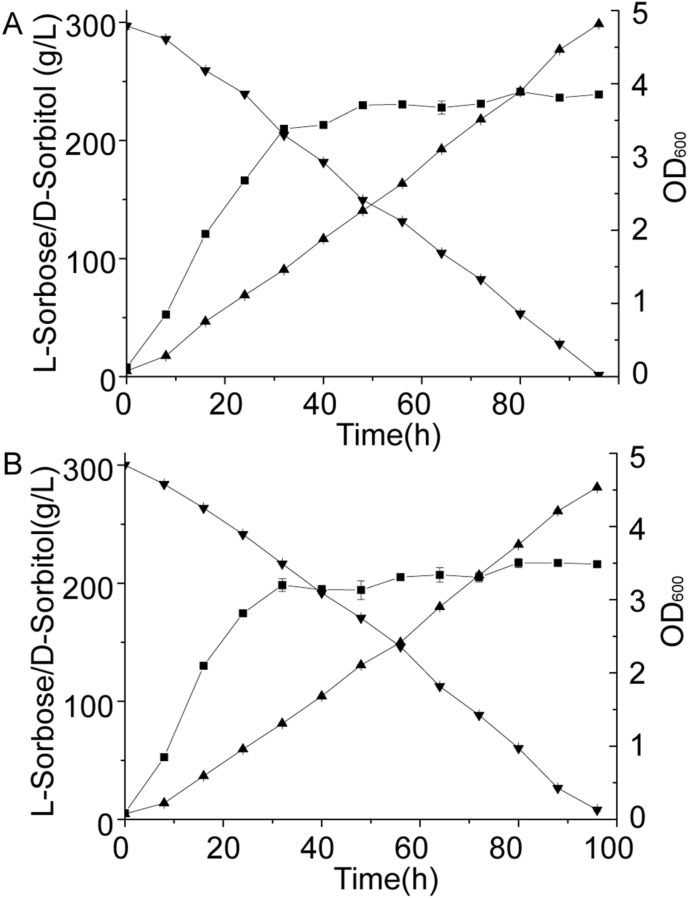
**Time course of****l****-sorbose production in the 5-L bioreactor**. (A) Time course for the production of l -sorbose by *G. oxydans* WSH-003 with p13-tet. (B) Time course for the production of l-sorbose by *G. oxydans* MD-16 with p13-tet-P_vhb_-Tat-*vhb*. Up-triangle, l-sorbose; down-triangle, D-sorbitol; square, OD_600_.

Based on the effects of VHb expression in the shaking flask, the relatively high-production strain expressing VHb in the periplasm (*G. oxydans* MD-16 with p13-tet-P_vhb_-Tat-*vhb*) was chosen to scale-up in a 5-L bioreactor. The *G. oxydans* strain harboring p13-tet was used as the control during the fermentation process (Fig. 4). The production of l-sorbose with *G. oxydans* MD-16 having p13-tet-P_2703_-Tat-*vhb* was 298.61 g/L with a conversion rate of 99.54%, while the control strain had a yield of 281.07 g/L with a conversion rate of 94.70%. Besides, the maximum OD_600_ of the engineered strain and the control strain was 3.85 and 3.50, respectively. More importantly, the *G. oxydans* MD-16 with p13-tet-P_2703_-Tat-*vhb* had fewer by-products in the fermentation broth, thus facilitating the downstream process.

## 4 Conclusions

In this study, the role of *G. oxydans* WSH-003 dehydrogenases in l-sorbose production was systematically investigated. The results proved that the engineering of dehydrogenases could further improve the titer and conversion rate of l-sorbose. By knocking out some dehydrogenase genes, the amounts of by-products of *G. oxydans* could be significantly reduced. Meanwhile, both the titer and the conversion rate of l-sorbose were improved. Additionally, by systematically knocking out dehydrogenases of *G. oxydans*, a high-performing strain achieved 149.46 g/L l-sorbose titer and 99.60% theoretical yield in the flask. By overexpressing *vhb* in *G. oxydans* MD-16, the titer of l-sorbose reached 298.61 g/L in a 5-L bioreactor. The systematic characterization and engineering of dehydrogenases in *G. oxydans* could also provide references for more efficient biosynthesis of other compounds in this strain.

## CRediT authorship contribution statement

**Li Liu:** Methodology, Investigation, Formal analysis, Writing – original draft. **Yue Chen:** Investigation, Writing – original draft, Validation. **Shiqin Yu:** Formal analysis, Writing – review & editing. **Jian Chen:** Funding acquisition. **Jingwen Zhou:** Supervision, Funding acquisition, Writing – review & editing.

## Declaration of competing interest

The authors declare that they have no competing interests.
