# Supplementary material for: Enhanced production of l-sorbose by systematic engineering of dehydrogenases in Gluconobacter oxydans
Source: Synth Syst Biotechnol. 2022 Mar 16;7(2):730–7. doi: 10.1016/j.synbio.2022.02.008 (PMC8927921; doi:10.1016/j.synbio.2022.02.008)
Supplement: Multimedia component 1 [file mmc1.docx]

**Supplementary Table**

**Supplementary Table 1. Primers used for knocking out single dehydrogenase.**

| **Primers** | **Sequence (5'-3')** |
| --- | --- |
| **Primers used for knocking out sorbitol dehydrogenase** | |
| 003-SLC-F | CTAACAGGCGCTTACGATGAGGTCT |
| 003-SLC-R | CTACCTGCACAAAACGTCCCGA |
| 003-SLC-CZ-F | GAGGCGACGAGACACTTCGAAGA |
| 003-SLC-CZ-R | TACAAGCCTCGGGTATCGCCATTC |
| 003-SLC- KU-F | CGATACCCGAGGCTTGTATGGCGGATCGGCGTAACGTAGC |
| 003-SLC-KU-R | TCGAAGTGTCTCGTCGCCTCCGTGTTTCTGCGGTGTGGTGA |
| 003BA1-F | GTTTCCGTGTTCAAATCTAGGCAG |
| 003BA1-R | GAACACATATCCAACCGAGAGCG |
| 003BA1-CZ-F | GGCTTCGTCTGGCTCGTACTTT |
| 003BA1-CZ-R | GACGTTCCGGAGCTTTGTGACT |
| 003BA1-KU-F | GTTCCGGAGCTTTGTGACTTGGCGGATCGGCGTAACGTAG |
| 003BA1-KU-R | AGTACGAGCCAGACGAAGCCCGTGTTTCTGCGGTGTGGTGAC |
| 003BA2-F | GCCACTACTTTCAGGATTCGGC |
| 003BA2-R | GTCCGGTTCACGCAGCATGATC |
| 003BA2-CZ-F | ATCCCGCTGCCCGACAGATACA |
| 003BA2-CZ-R | CTCCACTGGGGCGATAAAGTCC |
| 003BA2-KU-F | CCACTGGGGCGATAAAGTCCTGGCGGATCGGCGTAACGTAG |
| 003BA2-KU-R | TATCTGTCGGGCAGCGGGATCGTGTTTCTGCGGTGTGGTGAC |
| **Primers used for knocking out single dehydrogenase** | |
| 1-up-F | CCGAACTACAATCCAGATGCTCTCT |
| 1-up-R | GACTCATTCCAAATACCTC TCATTCCTTTCAGCGTGACGGTTTC |
| Kana-F | GAGGTATTTGGAATGAGTCGCCGTCA |
| 1-Kana-R | GAATACAAAAGAAAAAGGGC CCTTTCATAGAAGGCGGCGGT |
| 1-down-F | GCCCTTTTTCTTTTGTATTCAGGCCGT |
| 1-down-S-R | CGAACCGCAATTTCATCGTCAGACA |
| 2-up-F | ATGGTATTCCAGTCCTATGCGCTCTA |
| 2-up-R | GACTCATTCCAAATACCTC GAACTCAGGCGGCGAGGGG |
| 2-Kana-R | AGTTAATATGTCCGGGC CCTTTCATAGAAGGCGGCGGT |
| 2-down-F | GCCCGGACATATTAACTTCGGCGG |
| 2-down-R | ACAGCTATGACCATGATTA TTTCGATCATGTTCCGCCCCC |
| 3-up-F | TATCATGCGAACACAGGAACAACGATT |
| 3-up- R | GACTCATTCCAAATACCTCTGCAAAATCTTTTCCCTGTCTATGCTCA |
| 3-Kana-R | TGGAATTGTTATTTCAGCAA CCTTTCATAGAAGGCGGCGGT |
| 3-down-F | TTGCTGAAATAACAATTCCAACTGGCAG |
| 3-down-R | TGCAGAGACCGCCCCGTGAT |
| 4-up-F | TTGAAGAGTTCATGGACTACGCCAAGA |
| 4-up- R | GACTCATTCCAAATACCTC ACAATCTCTCTCTGCCAGCTCCG |
| 4-Kana- R | ACAGTTCTTTCAGCAGT CCTTTCATAGAAGGCGGCGGT |
| 4-down- F | ACTGCTGAAAGAACTGTCTCCCGAA |
| 4-down- R | CCGCGGTGACAGCAGGAATGAT |
| 5-up- F | ATCATCTGCCTTATCGGACAAG |
| 5-up-R | GACTCATTCCAAATACCTC TCTGAACAACATGAGATGAAGAGGC |
| 5-Kana -R | TTGAAGACGAAGACGATT CCTTTCATAGAAGGCGGCGGT |
| 5-down-F | AATCGTCTTCGTCTTCAAATCCTGGGT |
| 5-down-R | GCGGTTCTGCGAAGCCATTT |
| 6-up-F | GGGTATTTACCGATGTCAGGCCAGAT |
| 6-up-R | GACTCATTCCAAATACCTC TCACGCACTTTCAGGTAACCACGA |
| 6-Kana-R | CATAAATCCTGGTTCATCC CCTTTCATAGAAGGCGGCGGT |
| 6-down-F | GGATGAACCAGGATTTATGTACTGGCG |
| 6-down-R | GGTATGACCGCCAGCAATCAGG |
| 7-up-F | GTGGCAAAGTTCATTTCATTGCTTG |
| 7-up-R | GCGACTCATTCCAAATACCTCATTTCACCTTGGTGCGCTCAATGGCG |
| 7-Kana-R | TGATTGCACCACCGTGCTCTCCTTTCATAGAAGGCGGCGGTGGA |
| 7-down-F | AGAGCACGGTGGTGCAATCAAGCAT |
| 7-down-R | TGCAGATCACGCGCGTTACG |
| 8-up-F | CTATCACGCTCATCCTCATGGGG |
| 8-up-R | GACTCATTCCAAATACCTCCAATCTGGTGACGTTAAGCCTCAA |
| 8-Kana-R | GAACAGTTTCTACCCTGA CCTTTCATAGAAGGCGGCGGT |
| 8-down-F | TCAGGGTAGAAACTGTTCGGCGAT |
| 8-down-R | CATTCTTGTCCGTGCTCGCGCTAT |
| 9-up-F | CTCTCATGGCTGATACAATGCTGGC |
| 9-up-R | GACTCATTCCAAATACCTC GGTATGTGCTTTCTAAAGTCTCAGGACC |
| 9-Kana-R | TTACGTATGTATGTCGAGG CCTTTCATAGAAGGCGGCGGT |
| 9-down-F | CCTCGACATACATACGTAATACAGACGG |
| 9-down-R | TAGCAACGTCAAGGCGATTATCTGAA |
| 10-up-F | GGCCAGAAAATTCGATACGGACGA |
| 10-up-R | GACTCATTCCAAATACCTC GGAATTTCCTTTTCTGGGAAGACGGAT |
| 10-Kana-R | CATGATCAGTCTTCTTTACG CCTTTCATAGAAGGCGGCGGT |
| 10-down-F | CGTAAAGAAGACTGATCATGTGGTGAAAA |
| 10-down-R | ACTCCATCAGCAACGCTTTCACG |
| 11-up-F | GAGATGTTCCATGAATCTGGGGATAAGAG |
| 11-up-R | GACTCATTCCAAATACCTC TGACTGCTCCTGTATTCAGGGCG |
| 11-Kana- R | GACTGCCTTCAGAGAAA CCTTTCATAGAAGGCGGCGGT |
| 11-down- F | TTTCTCTGAAGGCAGTCTGGGAAAC |
| 11-down- R | GGATAACTCCAGTTCGACGAGCCA |
| 12-up-F | GCGTTCTGCCTGAGCTGT |
| 12-up-R | GACTCATTCCAAATACCTC CGCCGTTTCAGTTTCTGCT |
| 12-Kana- R | CGTAATCGAAACCAGTCATACCTTTCATAGAAGGCGGCGGT |
| 12-down- F | TATGACTGGTTTCGATTACG TTGTCGTTGGTGGCGGCT |
| 12-down- R | AATGGAACTGAAGATCCGGGCA |
| 13-up- F | ACGGAACTGCGTTTTGCGGATGA |
| 13-up- R | GACTCATTCCAAATACCTC GTCTGTAGTCTTCCTTTCCCGCAT |
| 13-Kana-R | GCGGAACACGATCCA CCTTTCATAGAAGGCGGCGGT |
| 13-down-F | TGGATCGTGTTCCGCTGCGG |
| 13-down-R | TGAAGCCCGACTGGCAATCGG |
| 14-up-F | GACAGGCTCAAAATAGTCTGCCGC |
| 14-up -R | GACTCATTCCAAATACCTC GAGTGCTCTCTGACCTCTCGAACC |
| 14-Kana-R | TGTCAGTCAAAAGCGTATCCTTTCATAGAAGGCGGCGGT |
| 14-down-F | ATACGCTTTTGACTGACAACGCGAA |
| 14-down-R | CGTTTCCCGGCGGGTCTGA |
| 15-up-F | CCTGATGAATAAAGTACAGCTTGGCGC |
| 15-up-R | GACTCATTCCAAATACCTC GGCGTATCCTGTCTGTCCATGCT |
| 15-Kana-R | TTCATGATGTCTGTATCCC CCTTTCATAGAAGGCGGCGGT |
| 15-down-F | GGGATACAGACATCATGAAACAGGTTGG |
| 15-down-R | TAGGCTTCGACAAGCGCAATCCG |
| 16-up-F | TTGGCCTCATTACGCCCTGGAACTT |
| 16-up-R | TTCATGATGTCTGTATCCC ATCAGGAGATCCAGTGCGTCCGTTT |
| 16-Kana-R | AAGCGCCTGAAAACATTGTCTCCTTTCATAGAAGGCGGCGGTGGAA |
| 16-down-F | AGACAATGTTTTCAGGCGCTTCCTCG |
| 16-down-R | CCAGCCGACCGTCTTCAATGTGCATCT |
| 17-up- F | TCTTGATATTAATGCCGCCATGTTCTCC |
| 17-up-R | GACTCATTCCAAATACCTC CGTTTTCAGCCTGCAATGACATTCG |
| 17-Kana-R | ATCCAGATCAAAGAAACGA CCTTTCATAGAAGGCGGCGGT |
| 17-down-F | TCGTTTCTTTGATCTGGATGCTTTAGGG |
| 17-down-S-R | GGCAGCCATGTCACAAAAACCC |
| 18-up- F | TGAAAGCCTATCTGGAAGACTGGCG |
| 18-up- R | GACTCATTCCAAATACCTC ACCCAACCGTCTCCTGTCG |
| 18-Kana-R | CTCTCAGAGAACGATCTG CCTTTCATAGAAGGCGGCGGT |
| 18-down-F | CAGATCGTTCTCTGAGAGGCGGTG |
| 18-down-R | ACGTTGTGGAAGTGTTGGCATGAT |
| 19-up-F | AGTCCTGGCATACTTATATTCCTTATACTGAT |
| 19-up- R | GACTCATTCCAAATACCTCCAGCCATCACGAAGTCAATGCTGC |
| 19-Kana-R | AGGGTTTTCAATGCCTTA CCTTTCATAGAAGGCGGCGGT |
| 19-down-F | TAAGGCATTGAAAACCCTTACGCCG |
| 19-down-R | CACCCATGGCAGCCCGTC |
| 20-up- F | GAAGCTTTCGTCATCATAGAGATAGGCTG |
| 20-up-R | GACTCATTCCAAATACCTC CAACCAGCGTCTCCAAAGGTTTCA |
| 20-Kana-R | GGTTCTCAGAGGTCATTTAT CCTTTCATAGAAGGCGGCGGT |
| 20-down-F | ATAAATGACCTCTGAGAACCTGCCCAT |
| 20-down-R | CGGTCGGAAATAGCCTTGCGG |
| 21-up-L-F | ATCATTCCCTTGATGAATACGGTCTGC |
| 21-up-R | GACTCATTCCAAATACCTC AAGGCCCGTCGTGCAATCAG |
| 21-Kana-R | CCTGAATTTATCGGGGAAT CCTTTCATAGAAGGCGGCGGT |
| 21-down-F | ATTCCCCGATAAATTCAGGGCTGAGG |
| 21-down-R | GTCAGAGGTCGCGCTTGCATAG |
| 22-up-F | CAGATCTCGTGGGTAAAGTCGCG |
| 22-up-R | GACTCATTCCAAATACCTCTTCCTATCGTGAAGCAATAATTGACGCG |
| 22-Kana-R | CTACTCTTTAATGAGAGGTAAT CCTTTCATAGAAGGCGGCGGT |
| 22-down- F | ATTACCTCTCATTAAAGAGTAGGAAAAAAAACGCA |
| 22-down- R | AGGTCGTAACAGGCAGTTTGTCC |
| 23-up- F | CGAAAGAAACAGGCATCGTTCCGC |
| 23-up- R | GACTCATTCCAAATACCTCGAGAGTGTCTCCTTGAGAACTGGTTTACG |
| 23-Kana- R | GGTATGTGCTTTCTAAAGTC CCTTTCATAGAAGGCGGCGGT |
| 23-down- F | GACTTTAGAAAGCACATACCATGTTTGCC |
| 23-down- R | TACGTATGTATGTCGAGGCGCAGG |
| 24-up- F | AACGAGGGTATCGATAAATGTCCGGA |
| 24-up- R | GACTCATTCCAAATACCTCCCTGTCTTTACCCTGTGACTGGAAAC |
| 24-Kana- R | CTGGTCAGAACATTTCAG CCTTTCATAGAAGGCGGCGGT |
| 24-down- F | CTGAAATGTTCTGACCAGCGCATCC |
| 24-down- R | CAATCGATGGATCGTCTGATGAGCAT |
| 25-up- F | ACACGACAGATAAGATCAGGAGACTGG |
| 25-up- R | GACTCATTCCAAATACCTC AGTGGTCTCCAGAACGAGGGTG |
| 25-Kana-R | ATGTTTTTTGGTCATGATCT CCTTTCATAGAAGGCGGCGGT |
| 25-down-F | AGATCATGACCAAAAAACATGCAGATGCC |
| 25-down-R | ATTGCTGACGGGATCATACGGTTTT |
| 26-up-F | GATCGTATCCTTTCGGATATTGCCAACG |
| 26-up-R | GACTCATTCCAAATACCTC GTCCTTACCTTTTCCTGAGCGTCAAT |
| 26-Kana-R | CAATGATGAGAACAGTGAG CCTTTCATAGAAGGCGGCGGT |
| 26-down-F | CTCACTGTTCTCATCATTGAAGATGATTTCCTG |
| 26-down-R | CCTGAGGGCAGCTTCGTTCG |
| 27-up-F | CAGGCTCAGGATGATAATGGAGCCC |
| 27-up-R | GACTCATTCCAAATACCTC GAATTTCAAAGCCTCCGCCCAAGA |
| 27-Kana-R | CTGATGGACGTATAAATGTC CCTTTCATAGAAGGCGGCGGT |
| 27-down-F | GACATTTATACGTCCATCAGTGCGGC |
| 27-down-R | CACAATTCGGCTGCCTTTTCCCATA |
| 28-up- F | GAAAACAACTGGGAACAGGCTGCC |
| 28-up- R | GACTCATTCCAAATACCTC GACAGTTTCCTCTTATCGGGCGC |
| 28-Kana-R | GAACTTGAAACCTTTTTCTG CCTTTCATAGAAGGCGGCGGT |
| 28-down-F | CAGAAAAAGGTTTCAAGTTCCTGCAGGC |
| 28-down-R | CAGAAGGATGCTGCGTGTGATGC |
| 29-up-L-F | GTTTCACACTTGGATTGATTGGCGGG |
| 29-up-L-R | GACTCATTCCAAATACCTC TCTCTCCCCCGTGCTTCAATATCA |
| 29-Kana-L -R | ATCACGTTTCTGTTTTAACA CCTTTCATAGAAGGCGGCGGT |
| 29-down-L-F | TGTTAAAACAGAAACGTGATGGATGTGTGT |
| 29-down-S-R | CCTGCATCGCATATCCGGAAGCATT |
| 30-up-L-F | AGTATTGTTCAGAGGCTTACCGTCAG |
| 30-up-L-R | GACTCATTCCAAATACCTCGTATATTCGTTAAGTATCTCACCTCGGTACTCC |
| 30-Kana-R | GAAAAGGAATTAACCAAACC CCTTTCATAGAAGGCGGCGGT |
| 30-down- F | GGTTTGGTTAATTCCTTTTCTTTAGA |
| 30-down- R | CATGGCTGAATTCCGCCCCAA |
| 31-up-L-F | TCTTCCTTCCAGAGATAAAAACCTGACCAG |
| 31-up-L-R | GACTCATTCCAAATACCTC GATGGTTCTTCCGAACGAAAGGCC |
| 31-Kana-R | ATGGTTTCAGACAATCATG CCTTTCATAGAAGGCGGCGGT |
| 31down-F | CATGATTGTCTGAAACCATCTCCAGGAC |
| 31down-R | GGCAACACCGAAACGACAGATAGAAA |
| 32-up-F | CATCGCTTACAGTACAAATACAAAACGGGC |
| 32-up-R | GACTCATTCCAAATACCTC GATGTTCCTAGAGCTGTTATTTGGTGCA |
| 32-Kana-R | TTTCAACAAAAAAGGGCA CCTTTCATAGAAGGCGGCGGT |
| 32-down-F | TGCCCTTTTTTGTTGAAACGGCTTTACA |
| 32-down-R | GGCAGGGGTTTCACACTTGGT |
| 33-up-L-F | GAAAAGACGATACATTGCACGGCTCC |
| 33-up-L-R | GACTCATTCCAAATACCTCGGGAGACCTCCATAAAACTCTCGACAG |
| 33-Kana-L -R | CCGTCAGTTATTTTCAGAC CCTTTCATAGAAGGCGGCGGT |
| 33-down-L-F | GTCTGAAAATAACTGACGGCAGCAAGT |
| 33-down-S-R | GGATTACTCCGGCGGGGCTT |
| 34-up-F | CAATAAAAATCCTGGCCATATACGTCGCA |
| 34-up-R | GACTCATTCCAAATACCTCGTACGGTGTCCCTTATAGAGTGCGC |
| 34-Kana-R | TAAAAAAGAAAATAGGGCGT CCTTTCATAGAAGGCGGCGGT |
| 34-down-F | ACGCCCTATTTTCTTTTTTAGGAAACTGC |
| 34-down-R | AACAGGGTTGGAGGGGAGCT |
| 35-up-F | AAAGCAGAAATCTACACGCTCATGCGG |
| 35-up-R | CGACTCATTCCAAATACCTCATTCACTATCCTGTGATGCTGTCTCAGTG |
| 35-Kana-R | CGCGGGCAGTGACACGGTTCCATCCTTTCATAGAAGGCGGCGGTGGAATC |
| 35-down-F | ATGGAACCGTGTCACTGCCCGCGG |
| 35-down-R | CGATCTGGACCGGGACCGC |
| 36-up-F | GTTTGTGCCTTCTGATACGAAGACGCT |
| 36-up-R | GACTCATTCCAAATACCTC TAAAATAGTACTTCAACATATTGGCATGAAAGC |
| 36-Kana-R | TATACTTGAAAGACCAGAGT CCTTTCATAGAAGGCGGCGGT |
| 36-down-F | ACTCTGGTCTTTCAAGTATAAAAAAACCCCGG |
| 36-down-R | AAGCCAGGCGCATAAAACTCG |
| 37-up-F | AGCTGAAATATAGCAAATCCAACAACGACT |
| 37-up-R | GACTCATTCCAAATACCTC GATCCGACTGTCCTTTTTGCAAAGACTG |
| 37-Kana-R | TTCATCTCAGATACCAGTC CCTTTCATAGAAGGCGGCGGT |
| 37-down-F | GACTGGTATCTGAGATGAAACAGGGATGG |
| 37-down- R | GGGTTGCCAGCCAGCGG |
| 38-up-F | CTTCTTCCTTTTATTCACGTTCTGCTGGT |
| 38-up-R | GACTCATTCCAAATACCTC CTGCCTTGACGCTATTTCGTTCTCAG |
| 38-Kana-R | AAGACTTAAGCCGTATCTT CCTTTCATAGAAGGCGGCGGT |
| 38-down-F | AAGATACGGCTTAAGTCTTCAAGGCAG |
| 38-down-R | CTCGACGTCCACTGGGGTCT |
| 39-up-F | CTCGATATTTTCATGAGCGCAGGCAA |
| 39-up-R | GACTCATTCCAAATACCTC TCATGCCTGCGAAAGATCATTGC |
| 39-Kana-R | GATGAGCATGAAGATCTTTT CCTTTCATAGAAGGCGGCGGT |
| 39-down-F | AAAAGATCTTCATGCTCATCCAGAGCTGA |
| 39-down-R | CATCAAAGTCGTAATCGGGCGCAT |
| 40-up-F | ATCAGGAGTTCAATACGATCCTGAGCA |
| 40-up-R | GACTCATTCCAAATACCTC TAACGCCTCCACCATCATTGACTG |
| 40-Kana-R | GCATATAGACCAGAGAAGTA CCTTTCATAGAAGGCGGCGGT |
| 40-down-F | TACTTCTCTGGTCTATATGCAGTGAAATAAATA |
| 40-down-R | GGATAACTCCAGTTCGACGAGCCA |
| 41-up-F | ACCTAATGTGATGATGGTTTCGTTCGG |
| 41-up-R | GACTCATTCCAAATACCTC GACACCTCTGTTCTTCAAAAAATGGAAACC |
| 41-Kana-R | CATGACATTTATTTCCGCT CCTTTCATAGAAGGCGGCGGT |
| 41-down-F | AGCGGAAATAAATGTCATGGTTCGCAC |
| 41-down-R | CGGTTTTCTTCAAGAGTGTCGGCT |
| 42-up-F | GGTATTATCGGTCTTTCAACCGCGCTC |
| 42-up-R | GACTCATTCCAAATACCTCGCTGCCGATATCCTCAATCGTCGTT |
| 42-Kana-R | GCCATCATTCAACACTAAA CCTTTCATAGAAGGCGGCGGT |
| 42-down-F | TTTAGTGTTGAATGATGGCGGACGAAAT |
| 42-down-R | CTGATATGAGGGCGTCTGCCG |
| 43-up-F | ACATGAGAACAGATATCGTGCCGCG |
| 43-up-R | GACTCATTCCAAATACCTC GATGCGTTTTTCCCACTGTTTGAAGT |
| 43-Kana-R | AAATCTGCCGTTTTAAGAG CCTTTCATAGAAGGCGGCGGT |
| 43-down-F | CTCTTAAAACGGCAGATTTTCTCCATTGAT |
| 43-down-R | CGAGCCAGTCATTTGCCAGACAC |
| 44-up-F | CAGAAGGCAAAACGATGGGTGAGC |
| 44-up-R | GACTCATTCCAAATACCTCGGGTAACCGAAAGGACTAATTCCGGAT |
| 44-Kana-R | AATAAGTGTGCTGTTTTCC CCTTTCATAGAAGGCGGCGGT |
| 44-down-F | GGAAAACAGCACACTTATTCCGTGAAG |
| 44-down-R | CGAAAGCGTCCCCTTCAACGGA |
| **Primers used for markerless knock-out dehydrogenase** | |
| upp-up-F | CAAACGGCAGAACTGGTCGTGAT |
| upp-up-R | GCCCACGGGATTACGTCGGCTATATTGTCCCGGGTCTGG |
| upp-down-F | ACAATATAGCCGACGTAATCCCGTGGGCGGCGGCCCATC |
| upp-down-R | GCAGAAGGAGGAAAGAATAAGCGTC |
| Pdnak-S-F | GATGACAGTTGGAGAGATCGGGCGG |
| Pdnak-S-R | GTACTCTTTGTCCTTTCAGCGGTTT |
| kana-F | TAAACGGACGCACTGGATCTCCTGATGAGGTATTTGGAATGAGTCGCCGTCA |
| kana-R | GTCAGTCTCCAGAAAATCTGAGGTTCCTTTCATAGAAGGCGGCGGTGGAA |
| upp-F | ATTCCACCGCCGCCTTCTATGAAAGGAACCTCAGATTTTCTGGAGACTGACCA |
| upp-R | CGAGGAAGCGCCTGAAAACATTGTCTTAACCGGCCATAAAACGGCATGGTAT |
| upp-kana-F | TGGCGGATCGGCGTAACGTAGC |
| upp-kana-R | CGTGTTTCTGCGGTGTGGTGAC |
| MD-1-F | TGAAAGGACAAAGAGTACATGAGTCAGGCGCTGCCG |
| MD-1-R | ATCTCTCCAACTGTCATCCGCCGTTTCAGTTTCTGCTGTG |
| MD-2-F | GAGATACTTAACGAATATACTGGCGGATCGGCGTAACGTAGC |
| MD-2-R | TCCAGTTAGTCCTTTCTAAACGTGTTTCTGCGGTGTGGTGAC |
| MD-3-F | CACCACACCGCAGAAACACGCAGATCGTTCTCTGAGAGGCGGT |
| MD-3-R | TACGTTACGCCGATCCGCCAACCCAACCGTCTCCTGTCG |
| MD-4-F | CACCACACCGCAGAAACACGCATCGCCAGCTTGCTTCCCCCA |
| MD-4-R | TACGTTACGCCGATCCGCCACGCCCCTTCTGTTAACTGGTG |
| MD-5-F | AGGGAAAAGATTTTGCATGGCGGATCGGCGTAACGTAGC |
| MD-5-R | TGGAATTGTTATTTCAGCAACGTGTTTCTGCGGTGTGGTGAC |
| MD-6-up-F | CTAACAGGCGCTTACGATGAGGTCT |
| MD-6-up-R | GAGGCGACGAGACACTTCGAAGA |
| MD-6-down-F | CTACCTGCACAAAACGTCCCGA |
| MD-6-down-R | TACAAGCCTCGGGTATCGCCATTC |
| MD-6-upp-kana-F | CGATACCCGAGGCTTGTATGGCGGATCGGCGTAACGTAGC |
| MD-6-upp-kana-R | TCGAAGTGTCTCGTCGCCTCCGTGTTTCTGCGGTGTGGTGA |
| MD-7-up-F | ATCATGTCGCCTGCAAATCGTTAT |
| MD-7-up-R | TTGGAATTGTGGGGGTGGGAGAT |
| MD-7-down-F | AGCGGCTTCGGCACAAAGTCC |
| MD-7-down-R | CACAAATCTCCGGGAAAACTGCCAT |
| MD-7upp-kana-F | AGTTTTCCCGGAGATTTGTG TGGCGGATCGGCGTAACGTAGC |
| MD-7-upp-kana-R | CCCACCCCCACAATTCCAA CGTGTTTCTGCGGTGTGGTGAC |
| MD-8-F | AGGTATTTGGAATGAGTCTGGCGGATCGGCGTAACGTAGC |
| MD-8-R | CCATGACATTTATTTCCGCTCGTGTTTCTGCGGTGTGGTGAC |
| MD-9-F | AGGTATTTGGAATGAGTCTGGCGGATCGGCGTAACGTAGC |
| MD-9-R | TGGTTTCAGACAATCATGCGTGTTTCTGCGGTGTGGTGAC |
| MD-10-F | ATTAGTCCTTTCGGTTACCCTGGCGGATCGGCGTAACGTAGC |
| MD-10-R | GAATAAGTGTGCTGTTTTCCCGTGTTTCTGCGGTGTGGTGAC |
| MD-11-F | ATGATCTTTCGCAGGCATGATGGCGGATCGGCGTAACGTAG |
| MD-11-R | GATGAGCATGAAGATCTTTTCGTGTTTCTGCGGTGTGGTGAC |
| MD-12-F | GAAATAGCGTCAAGGCAGTGGCGGATCGGCGTAACGTAGC |
| MD-12-R | AAGACTTAAGCCGTATCTTCGTGTTTCTGCGGTGTGGTGAC |
| MD-13-F | AGGTATTTGGAATGAGTCTGGCGGATCGGCGTAACGTAGC |
| MD-13-R | CGGATATCGTTATTTCGTCCCGTGTTTCTGCGGTGTGGTGAC |
| MD-14-F | CCCTCGTTCTGGAGACCACTTGGCGGATCGGCGTAACGTAGC |
| MD-14-R | ATGTTTTTTGGTCATGATCTCGTGTTTCTGCGGTGTGGTGA |
| MD-15-up-F | GCAGCCCAACCCAGCCGATGAT |
| MD-15-up-R | CCTTCAATATGGTACGCGCTCCTG |
| MD-15-down-F | CATTCTTTCAAGGGCGCAGACCAT |
| MD-15-down-R | AGCTGTAACCATTCAAGGCTGGCG |
| MD-15-upp-kana-F | AGCGCGTACCATATTGAAGG TGGCGGATCGGCGTAACG |
| MD-15-upp-kana-R | AGCCTTGAATGGTTACAGCTCGTGTTTCTGCGGTGTGGTGAC |
| MD-16-up-F | GAAAAGATCCCGGACAGATTGGC |
| MD-16-up-R | GGAAGTCGCGCAATGATCATGTCC |
| MD-16-down-F | CGTAGTTGGCCGTCAGGTTGAAAT |
| MD-16-down-R | AGAATTTCAGCCGTCATAGTGGTG |
| MD-16-upp-kana-F | ATGATCATTGCGCGACTTCC TGGCGGATCGGCGTAACG |
| MD-16-upp-kana-R | ACTATGACGGCTGAAATTCTCGTGTTTCTGCGGTGTGGTGAC |
| MD-17-F | GCAAAAAGGACAGTCGGATCTGGCGGATCGGCGTAACGTAGC |
| MD-17-R | CGCGTATTTCCATCCCTGTCGTGTTTCTGCGGTGTGGTGAC |
| MD-18-up-F | AGTCACCTGATCTGGATTGGCG |
| MD-18-up-R | TCCTGCAATGCCTCAATCGTTC |
| MD-18-down-F | TTCCATGCAAGAGAAGGGGACC |
| MD-18-down-R | TGAATCAGGGGTGCAGACTGG |
| MD-18-upp-kana-F | ACGATTGAGGCATTGCAGGATGGCGGATCGGCGTAACG |
| MD-18-upp-kana-R | TCTGCACCCCTGATTCA CGTGTTTCTGCGGTGTGGTGAC |
| MD-19-F | GTTTCAACAAAAAAGGGCACGTGTTTCTGCGGTGTGGTGAC |
| MD-19-R | AGGTATTTGGAATGAGTCTGGCGGATCGGCGTAACGTAGC |
| MD-20-up-F | TGCTCATCCACCAGATACCCGAT |
| MD-20-up-R | GCTTGTGCGTCATATAGTCGTGGAAA |
| MD-20-down-F | TCTGCACTAAGAGTCGCCGCGTT |
| MD-20-down-R | GGAGGGAGGCCGAATGCACGATG |
| MD-20-upp-kana-F | CGACTATATGACGCACAAGC TGGCGGATCGGCGTAACG |
| MD-20-upp-kana-R | CGTGCATTCGGCCTCCCTCCCGTGTTTCTGCGGTGTGGTGAC |
| MD-21-up-F | CTAATTGTGCGATTGCTTTTGCTCC |
| MD-21-up-R | AATGCATGGAAGCAGGTTTTCG |
| MD-21-down-F | ATTTTTGACAGTTCTCCGGCCGAA |
| MD-21-down-R | TTGATACGATGGGGAACATGTTTGC |
| MD-21-upp-kana-F | AAAACCTGCTTCCATGCATT TGGCGGATCGGCGTAACG |
| MD-21-upp-kana-R | CATGTTCCCCATCGTATCAA CGTGTTTCTGCGGTGTGGTGAC |
| MD-22up-F | CGTCACCGTCGCAGGTGGCCTG |
| MD-22-up-R | GAGAGTGTCTCCTTGAGAACTGGTTTACGA |
| MD-22-down-F | GTTCTCAAGGAGACACTCTCTGGCGGATCGGCGTAACGTAGCCGG |
| MD-22-down-R | ATTACGTATGTATGTCGAGGCGTGTTTCTGCGGTGTGGTGACAA |
| MD-22-upp-kana-F | CCTCGACATACATACGTAATACAGACGGATAA |
| MD-22-upp-kana-R | AATGACTCAGCATAATGCGGCCAT |
| MD-23-up-F | GTCACTATAAATTCGGTTTTGCCTACG |
| MD-23-up-R | ACCTGGATGGCAACCGGTAATA |
| MD-23-down-F | AAGAGCGTGAAGCCCCATAAAAC |
| MD-23-down-R | GTTGTGAGGCATGTCCGTGG |
| MD-23-upp-kana-F | ACCGGTTGCCATCCAGGTAG TGGCGGATCGGCGTAACG |
| MD-23-upp-kana-R | CGGACATGCCTCACAAC CGTGTTTCTGCGGTGTGGTGAC |
| MD-24-up-F | TTTCCTGTCCCACCTCAACCGA |
| MD-24-up-R | AACTGACACGTCTACCAGCAACCAT |
| MD-24-down-F | CACGATGAGCGCCTTAGAGTTTCAA |
| MD-24-down-R | CGGCTGCTCCTGCTCATAGTTTTG |
| MD-24-upp-kana-F | TGCTGGTAGACGTGTCAGTTTGGCGGATCGGCGTAACGTAGC |
| MD-24-upp-kana-R | ACTCTAAGGCGCTCATCGTGCGTGTTTCTGCGGTGTGGTGAC |
| MD-25-F | AGGTATTTGGAATGAGTCTGGCGGATCGGCGTAACGTAGC |
| MD-25-R | CGAAGTTAATATGTCCGGGCCGTGTTTCTGCGGTGTGGTGAC |
| MD-26-F | AGGTATTTGGAATGAGTCTGGCGGATCGGCGTAACGTAGC |
| MD-26-R | CATGATCAGTCTTCTTTACG CGTGTTTCTGCGGTGTGGTGA |
| MD-27-F | TTCATCTCATGTTGTTCAGATGGCGGATCGGCGTAACGTAGC |
| MD-27-R | CAATGAGCGTGACCCAGGATCGTGTTTCTGCGGTGTGGTGAC |
| **Primers used for overexpression *vhb*** | |
| P13-CZ-F | TAATCATGGTCATAGCTGTTTCCTGT |
| P13-CZ-F | TATACTGTATGGGAACGGAAGACCT |
| P2703 -F | TTCCGTTCCCATACAGTATATGGTAGCAGATGTGGAGAAATATCCGC |
| P2703-R | TGCGTAATCCTTTCATAACACCATTCG |
| PVHb-F | TGTTATGAAAGGATTACGCATAAATTCAATGTGAAAAAGCTTACAGGACG |
| VHb-ATG-F | TGTTATGAAAGGATTACGCAATGTTAGACCAGCAAACCATTAACATCATC |
| VHb-R | AACAGCTATGACCATGATTAGGCAATATTTGTCCCAAGTTTTGGCC |
| P2703-TAT-F | TGTTATGAAAGGATTACGCAATGAACAATAACGATCTCTTTCAGGCATCACG |
| PVHb-TAT-F | ATGAACTTAAGGAAGACCCTCATGAACAATAACGATCTCTTTCAGGCATCACG |
| Tat-R | TTATTATGGTTTTGCCATACATACGTCGCGGCGTTAACAATGACGG |
